# Clinical benefit of various HER2-directed regimens in gastrointestinal malignancies: a retrospective cohort study

**DOI:** 10.1186/s12885-026-16309-3

**Published:** 2026-06-10

**Authors:** Vishesh Khanna, Swetha Vadde, A. Solomon Henry, Tyler P. Johnson, Shagufta Shaheen, Gregory M. Heestand, Thomas Holden, Curtis R. Chong, Lipika Goyal, George A. Fisher, Christopher T. Chen

**Affiliations:** 1 Division of Oncology, Department of Medicine, Stanford University School of Medicine, Stanford, CA United States; 2 Research Informatics Center, Department of Medicine, Stanford University School of Medicine, Stanford, CA United States

**Keywords:** HER2, Gastrointestinal malignancies, Anti-HER2 therapy, Treatment sequencing

## Abstract

**Background:**

Multiple drugs targeting the human epidermal growth factor receptor-2 (HER2) have shown clinical activity in gastrointestinal (GI) cancers. How to best integrate these various options in clinical practice alongside conventional therapy, however, is unclear.

**Methods:**

We assessed the clinical outcomes of 103 patients with advanced HER2-altered GI malignancies who received ≥ 1 line of anti-HER2 therapy at our institution between 2010 and 2023.

**Results:**

Next-generation sequencing (NGS) detected an *ERBB2* amplification in 66% (27/41) of tumors that were HER2 + by IHC/ISH. Conversely, all patients with *ERBB2* amplification were HER2 + by IHC. In the second-line and beyond, trastuzumab plus pertuzumab had the longest median time to treatment discontinuation (7.9 months), followed by trastuzumab deruxtecan (5.0 months). Among 25 patients who received ≥ 2 anti-HER2 agents, 24% had more durable clinical benefit to their second-line HER2 therapy compared to their first, as measured by the growth modulation index.

**Conclusions:**

Multimodal HER2 testing is needed to accurately identify candidates for HER2-targeted treatment, as NGS alone misses a significant proportion of cases. Patients who develop resistance to one anti-HER2 agent may still achieve benefit from subsequent HER2-directed regimens. Early and serial treatment with HER2-directed agents should be considered for patients with HER2 + GI cancers.

**Supplementary Information:**

The online version contains supplementary material available at 10.1186/s12885-026-16309-3.

## Background

The human epidermal growth factor receptor 2 (HER2) is a validated oncogenic driver and therapeutic target across many cancer types [1–3]. In gastrointestinal (GI) malignancies, multiple classes of anti-HER2 therapies have gained regulatory approval, including monoclonal antibodies, tyrosine kinase inhibitors (TKIs), and antibody-drug conjugates (ADCs) [3–5]. Most clinical trials of novel HER2-directed therapies in GI cancers excluded patients treated with prior anti-HER2 agents, leaving the relative efficacy of follow-on HER2 blockade largely uncertain. For example, the HERACLES (trastuzumab plus lapatinib in colorectal cancer [CRC]) and the MyPathway (trastuzumab plus pertuzumab in advanced solid tumors) trials enrolled only HER2-targeted therapy-naïve patients [6, 7]. DESTINY-Gastric01, assessing trastuzumab deruxtecan (T-DXd) in gastroesophageal cancers, enrolled patients with prior trastuzumab exposure but did not compare outcomes by prior anti-HER2 therapy [8]. DESTINY-CRC01 similarly permitted prior anti-HER2 exposure, but did report this subgroup analysis: in HER2+ patients with prior HER2-targeted therapy, T-DXd achieved an objective response rate of 44%, comparable to the 46% rate observed in HER2-targeted therapy-naïve patients [9]. However, these trials were not designed to directly compare outcomes by prior anti-HER2 exposure, and real-world data on how to optimally sequence the growing array of HER2-directed agents across multiple lines of therapy remain limited.

Adding to this challenge is the lack of standardization in HER2 biomarker assessment across GI tumors. While immunohistochemistry (IHC), in situ hybridization (ISH), and next-generation sequencing (NGS) assessments of HER2 status are typically concordant in breast cancer, their alignment in GI tumors is less well characterized, leading to potential discrepancies that may impact patient selection and treatment decisions [10, 11]. For instance, previous data in gastroesophageal cancers have shown that a significant subset of HER2-positive tumors by IHC lack detectable *ERBB2* amplification on NGS [12]. These discrepancies raise concerns about the misclassification of patients, which may result in excluding truly eligible patients from receiving HER2-targeted therapy or leading to inaccurate treatment selection based on discordant biomarker assessments.

Given the rapidly evolving landscape of HER2-targeted agents and the heterogeneity of HER2 biomarker testing in GI malignancies, additional data are needed to guide patient selection and treatment strategies across multiple lines of therapy. In this study, we examine real-world utilization patterns and clinical outcomes associated with anti-HER2 agents in patients with advanced HER2-altered GI malignancies at a single institution.

## Methods

### Study population

This study included patients with advanced (metastatic or locally advanced unresectable) GI malignancies who received at least one HER2-directed therapy between January 1, 2010 – December 31, 2023. Study subjects were identified through the Stanford Cancer Institute Registry Database. HER2-directed therapy may have either been given as part of a patient’s initial systemic therapy regimen or in later lines of treatment. A full distribution of treatments received is shown in Supplementary Table 1. Included subjects also must have had a HER2/*ERBB2* alteration, defined as either (1) *ERBB2* amplification on NGS, (2) *ERBB2* amplification by ISH, (3) *ERBB2* mutation by NGS, (4) HER2 3 + by IHC, or (5) HER2 2 + IHC along with a positive ISH result. This cohort represents a subset of patients with HER2 + GI malignancies seen at our institution, limited to those with HER2 molecular and/or IHC testing performed at Stanford or available in the electronic medical record (EMR). Patients with HER2 testing performed externally and not available in the EMR were excluded.

### Pathologic and molecular analyses

All HER2 IHC and ISH assays were performed at Stanford’s CLIA-certified Clinical Pathology Laboratory on formalin-fixed paraffin-embedded (FFPE) tumor tissue, with fixation times following routine academic laboratory workflow (typically 6–72 h). HER2 IHC was performed using the 4B5 rabbit monoclonal antibody as part of the FDA-approved Ventana PATHWAY HER2 (4B5) kit, applied according to manufacturer instructions on an automated Ventana BenchMark platform (BenchMark XT prior to July 2017, BenchMark ULTRA from July 2017 onward). HER2 ISH was performed using the PathVysion HER-2 DNA Probe Kit (dual-color fluorescence in situ hybridization). HER2 positivity was defined as IHC 3 + or IHC 2 + with a positive ISH test result, using ToGA/CAP scoring criteria for gastric, gastroesophageal junction, and esophageal adenocarcinomas [13]. For non-gastroesophageal GI tumors (colorectal, biliary, pancreatic, and small bowel adenocarcinomas), tumor-specific HER2 scoring guidelines were not uniformly available during the study period, and cases were scored using criteria adapted from gastric or breast HER2 guidelines according to contemporaneous clinical pathology practice. IHC and ISH slides were interpreted visually by the signing-out board-certified pathologist. Borderline or difficult cases were reviewed at a multidisciplinary GI subspecialty consensus conference or by a second GI pathologist per routine laboratory practice.

Tumor next-generation sequencing (NGS) analysis was performed using Foundation CDx in 30/57 (53%) patients, Stanford’s internal Solid Tumor Actionable Mutation Panel (STAMP) in 21/57 (37%) patients, and other commercially available targeted sequencing panels in the remaining 6 (11%) cases. NGS reports were reviewed to identify concurrent mutations present in patients undergoing tumor NGS.

### Clinical outcomes

Time to treatment discontinuation (TTD) was defined as the duration between the treatment start date and the date of treatment discontinuation for any reason including progression, toxicity, death, or loss of follow-up. TTD calculations focused on (1) anti-HER2 agents received in the second-line of overall systemic therapy and beyond to exclude the effect of first-line cytotoxic chemotherapy in contributing to clinical benefit and (2) therapies received by 5 or more patients in the cohort. This resulted in the exclusion of TTD data for 60 patients receiving trastuzumab in combination with chemotherapy as frontline treatment for gastroesophageal adenocarcinoma as well as one patient who received a HER2 TKI in combination with chemotherapy.

In patients who received anti-HER2 agents and who underwent computed tomography (CT) or positron emission tomography-CT (PET-CT) scans for assessment of radiographic response, all scans were reviewed, and clinical radiographic response rates were calculated as the percentage of patients whose clinical notes and CT/PET-CT imaging reports noted a “response” or “response to treatment.” Stable disease was similarly defined by radiology reports and clinical notes stating no significant change in disease burden compared to prior imaging.

Time-to-event analysis were performed using the Kaplan-Meier method. Growth modulation indices (GMI) were calculated by dividing the time to treatment discontinuation (TTD) for the drug of interest by the TTD for the immediately preceding therapy. Patients who received HER2-targeted therapy in their first systemic treatment line were excluded from GMI analysis. All statistical analyses were performed using R statistical software.

## Results

### Patient characteristics and HER2 testing

A total of 103 patients with advanced HER2-altered GI malignancies met study criteria. The median age at diagnosis was 60 years (range: 19–81) and 61% were male (Table 1). The majority of patients (42%) identified as Caucasian, followed by Asian (29%). Most patients (81%) had Stage IV disease at diagnosis, with gastric (30%), esophageal (20%), and CRC (17%) representing the most common histologies.

**Table 1:** Patient demographics and clinical characteristics (*N* = 103)

Age (y) – median [range]	60 (19–81)
Sex – number (%)	
Female	40 (39%)
Male	63 (61%)
Race/Ethnicity	
White/Caucasian	43 (42%)
Asian/Pacific Islander	30 (29%)
Hispanic/Latino	20 (19%)
African American	4 (4%)
Unknown/Other	6 (6%)
Tumor Histology	
Gastric adenocarcinoma	31 (30%)
Esophageal adenocarcinoma	21 (20%)
Colorectal adenocarcinoma	18 (17%)
Gastroesophageal junction adenocarcinoma	15 (15%)
Biliary tract cancer	12 (12%)
Pancreatic adenocarcinoma	3 (3%)
Small bowel adenocarcinoma	3 (3%)
Disease Status at Diagnosis	
Localized/locally advanced (Stage I-III)	20 (19%)
Metastatic (Stage IV)	83 (81%)
HER2 expression by IHC	
3+	77 (75%)
2+	11 (11%)
1+	2 (2%)
0	4 (4%)
Unknown/missing	9 (9%)
*ERBB2* amplification by ISH	
Positive	38 (37%)
Negative	6 (6%)
Unknown/missing	59 (57%)
*ERBB2* alteration by NGS	
Amplification	33 (31%)
Wild type	16 (16%)
Extracellular domain (S310F)	3 (3%)
Kinase domain (T733I, G776V, L841C)	2 (2%)
Amplification + extracellular domain (S310F)	2 (2%)
Transmembrane domain (V659E)	1 (1%)
Unknown/missing	46 (45%)
*KRAS* mutation status	
Wild-type	51 (50%)
Mutated	9 (9%)
Unknown/missing	43 (42%)
Lines of anticancer therapy prior to first HER2 agent	
0^a^	61 (59%)
1	18 (17%)
2	16 (16%)
≥ 3^b^	8 (8%)
# of Lines of HER2-directed Therapy Received	
1	77 (75%)
2	16 (16%)
≥ 3^c^	10 (10%)
Percentage of Patients Receiving Specific HER2 Drug	
Trastuzumab^d^	82 (80%)
Trastuzumab deruxtecan	21 (20%)
Trastuzumab + pertuzumab	20 (19%)
HER2 TKI^e^	12 (12%)
Ado-trastuzumab emtansine	7 (7%)
Investigational product	4 (4%)

HER2 TKIs administered included lapatinib, neratinib, and tucatinib. Four patients received a HER2 TKI with chemotherapy, 4 with trastuzumab, 3 as monotherapy, and 1 with anti-PD-1 therapy

*Abbreviations*: *IHC* immunohistochemistry, *ISH* in situ hybridization, *NGS* next-generation sequencing

^a^ First-line anti-HER2 therapy (*N* = 61) was administered almost exclusively to patients with gastric, gastroesophageal junction, or esophageal adenocarcinoma (60/61) in combination with platinum-based chemotherapy; one additional patient with colorectal adenocarcinoma received a HER2 TKI (lapatinib) in combination with capecitabine as first-line therapy (see Supplementary Table 1)

^b^ Five patients received their 1st anti-HER2 agent in the 4th line; 3 in the 5th line

^c^ Among 10 patients who received 3 + lines of HER2-directed therapy, six received 3 lines, two received 4 lines, and one patient each received 5 and 6 lines

^d^ Three patients received single-agent trastuzumab as a 3rd (*N* = 2) or 4th line (*N* = 1) systemic therapy

^e^ Four patients received HER2 TKI with chemotherapy, 4 with trastuzumab, 3 as monotherapy, and 1 with anti-PD-1 therapy

HER2 status was assessed by IHC in 94/103 cases (91%), of whom 88 (86%) were IHC 3 + or 2+ (Table 1). Nine cases did not undergo IHC testing – five had only NGS, and 4 had NGS plus ISH (Supplementary Table 2). Of the 57 patients with NGS data, *ERBB2* amplification was present in 61% (35/57), and six (11%) harbored *ERBB2* point mutations (Table 1; Supplementary Fig. 1). Two cases had both *ERBB2* amplification and an S310F variant.

**Fig. 1:**
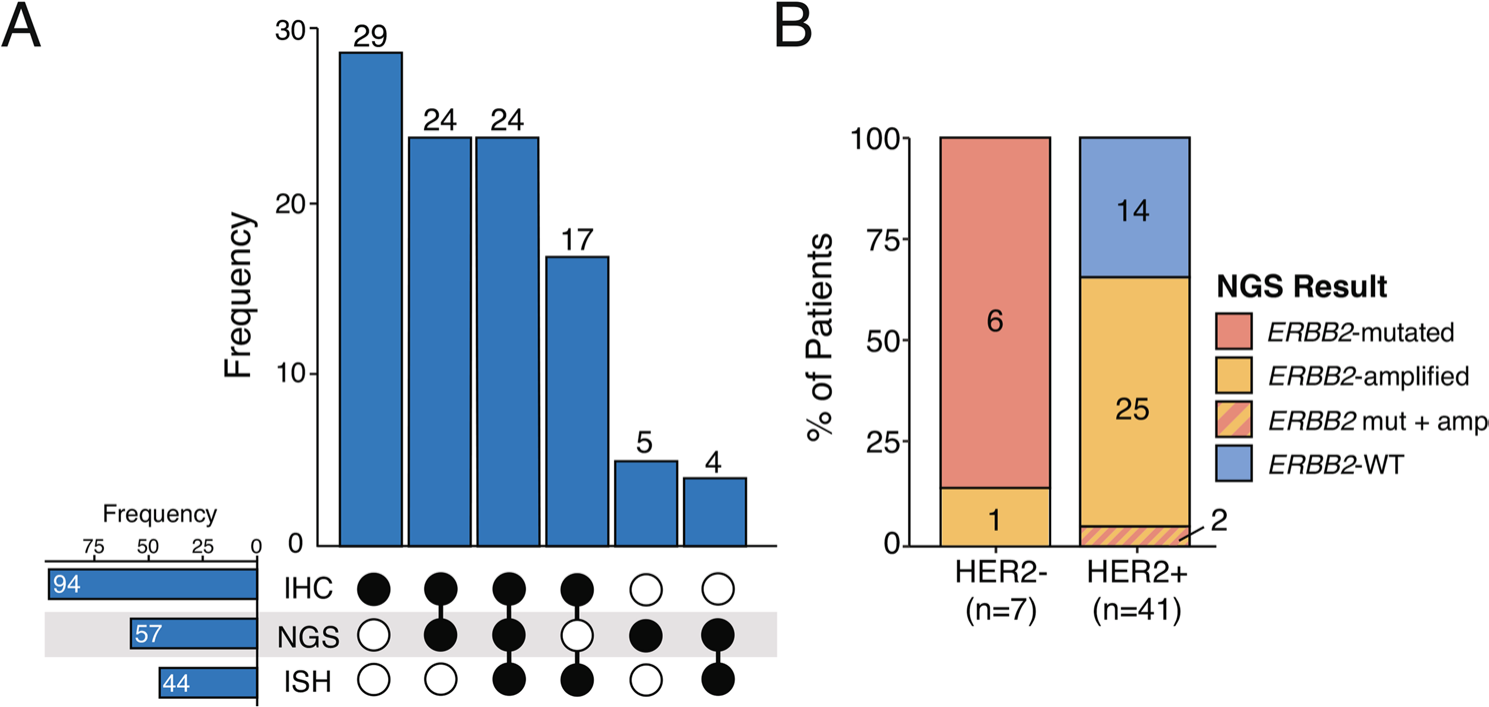
Summary of HER2 testing in the study cohort. **A** Breakdown of HER2 testing modalities for each of the 103 patients in the cohort. **B** Associations between HER2 testing results by traditional IHC/ISH based methods and targeted next-generation sequencing (NGS) (*N* = 48). IHC: immunohistochemistry. ISH: in situ hybridization. Mut + amp = co-mutated and amplified

Forty-eight patients had concurrent NGS and IHC results available (Fig. 1A), of whom 41 (85%) were IHC 3 + or IHC 2+/ISH+. Among these 41 patients with IHC/ISH+ tumors, *ERBB2* amplification was detected in 66% (27/41), two of whom were *ERBB2* co-amplified/mutated. The remaining 14 (34%) were *ERBB2* wild type by NGS (Fig. 1B), suggesting that a substantial proportion of cases that are positive by IHC/ISH-based testing may be missed by NGS alone. Conversely, among the 28 patients who were *ERBB2*-amplified and evaluable by IHC, 26 (93%) were IHC 3+. The remaining two were IHC 2+; one of these cases was ISH + and thus classified as HER2+, while the other lacked ISH testing and was considered HER2-negative. ISH results generally aligned with IHC and NGS, with 94% (32/34) of ISH-positive tumors also classified as IHC 2+/3 + and 91% (10/11) of NGS-amplified cases being ISH-positive (Supplementary Fig. 1). Notably, the rate of *ERBB2* amplification detection among HER2 + IHC/ISH cases was similar between the two major NGS platforms used (Foundation CDx: 13/23, 57%; STAMP: 9/13, 69%; Fisher exact test *p* = 0.50), with the IHC/NGS discordance observed at comparable rates across platforms (Supplementary Table 3).

### Use of HER2-directed therapy

**Fig. 2:**
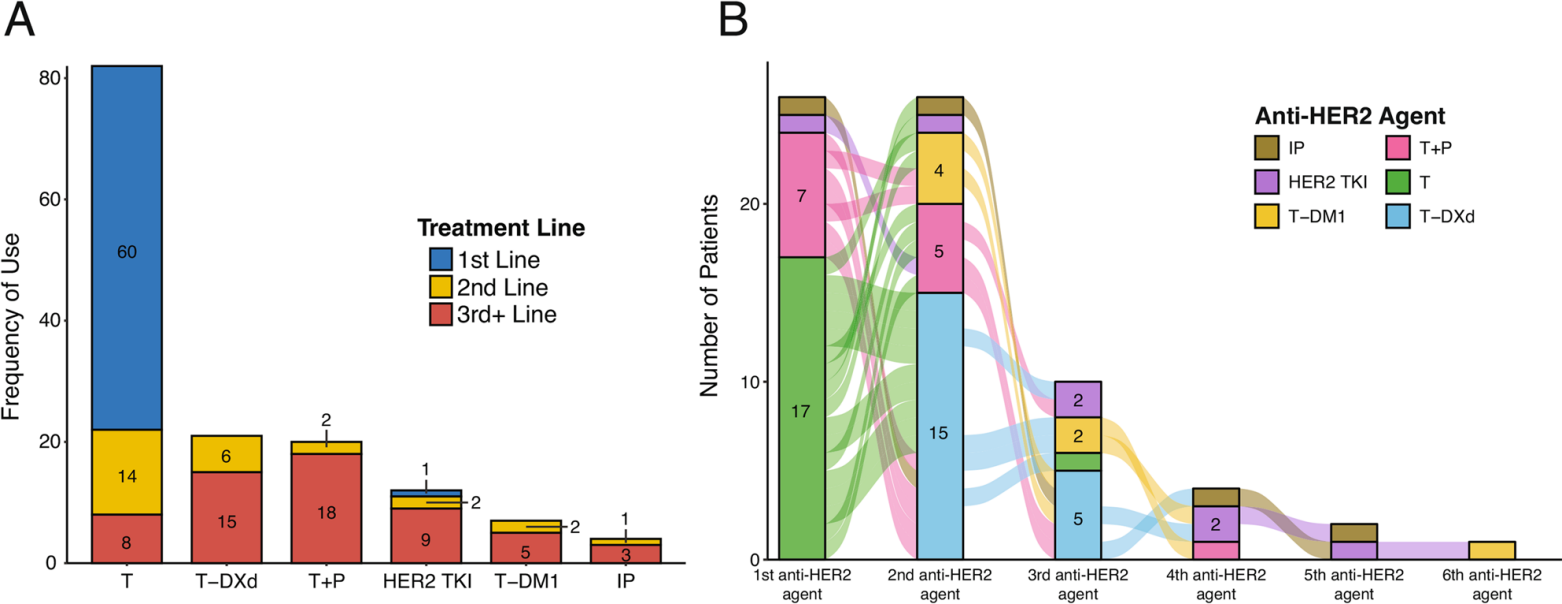
Patterns of Use of HER2-directed agents. **A** Bar plot showing the frequency with which each anti-HER2 agent was utilized in the study cohort. The color of each bar represents the line of treatment in which the corresponding anti-HER2 agent was received. **B** Alluvial plot displaying the sequence of how anti-HER2 agents were administered to patients receiving > 1 HER2 drug (*N* = 26 patients represented). Bars without numbers shown inside represent a single patient

Overall, 61/103 (59%) received HER2-directed therapy in the first-line setting, almost exclusively with trastuzumab (60/61; 98%) (Fig. 2A). The remaining 42 patients received their first HER2-directed agent in the second line or beyond (Fig. 2A). Most patients (77/103; 75%) received one line of HER2-directed therapy, whereas 26 patients (25%) received two or more lines. Trastuzumab was the most commonly used agent (82/103; 80%), followed by trastuzumab deruxtecan (T-DXd; 20%) and trastuzumab plus pertuzumab (T + P; 19%) (Fig. 2A). HER2 TKIs were administered to 12 patients, primarily in combination with chemotherapy or trastuzumab, and seven (7%) patients received ado-trastuzumab emtansine (T-DM1). HER2-directed investigational products (IP) were given to 4 (4%) patients. Among the 26 patients (25%) who received more than one line of HER2-directed therapy, trastuzumab was used the most frequently, with most transitioning to T + P or T-DXd as their next anti-HER2 agent (Fig. 2B). All seven patients who received T + P as their initial anti-HER2 regimen later switched to T-DXd.

### Clinical benefit of HER2-directed therapies

To exclude the effect of first-line cytotoxic chemotherapy, we examined the clinical benefit to HER2 regimens (T, T + P, T-DM1, T-DXd, TKIs) given in the second-line or beyond. Radiographic response assessments were available for 84 patients. Of 21 patients assessed for response to trastuzumab, 5 (24%) achieved documented radiographic reduction, all in combination with chemotherapy (Table 2). T-DXd also demonstrated a 24% rate of reduction among 21 patients (5/21). T + P had a 20% rate of reduction (4/20), whereas T-DM1 elicited no radiographic reduction (0/7). HER2 TKIs had an 18% rate of reduction (2/11), while one of four patients receiving investigational products achieved radiographic reduction.

**Table 2:** Clinical activity of HER2-directed treatments excluding 1st line treatments

Anti-HER2 Agent	Number of patients^a^	Median (95% CI) TTD (months)	GMI ≥ 1.33 (%)^b^	TTD ≥ 6 months & GMI ≥ 1.33 (%)	Rate of Radiographic Reduction
Trastuzumab	22	3.3 (2.5–8.3)	32% (6/19)^1^	26% (5/19)^1^	24% (5/21)^1^
T-DXd	21	5.0 (3.5–8.8)	24% (5/21)	14% (3/21)	24% (5/21)
T + P	20	7.9 (2.7–12.8)	53% (9/17)	53% (9/17)	20% (4/20)
HER2 TKI	11	3.5 (2.23 - NR)	36% (4/11)	27% (3/11)	18% (2/11)
T-DM1	7	1.6 (1.3 - NR)	0% (0/7)	0% (0/7)	0% (0/7)
IP	4	4.8 (1.2 - NR)	25% (1/4)	25% (1/4)	25% (1/4)

Patients without these data were excluded from the respective denominators

*NR* not reached, *TTD* time to treatment discontinuation (see main text for definition), *GMI* growth modulation index (see main text for definition)

^a^Total exceeds *n* = 81 because some patients received more than one anti-HER2 agent in the 2 L+ setting

^b^GMI calculation requires evaluable TTD on the immediately preceding line; response assessment requires imaging

^1^There were no patients receiving trastuzumab monotherapy who achieved a TTD ≥ 6 months, a GMI ≥ 1.33, or a clinical radiographic response – all were achieved in combination with chemotherapy

**Fig. 3:**
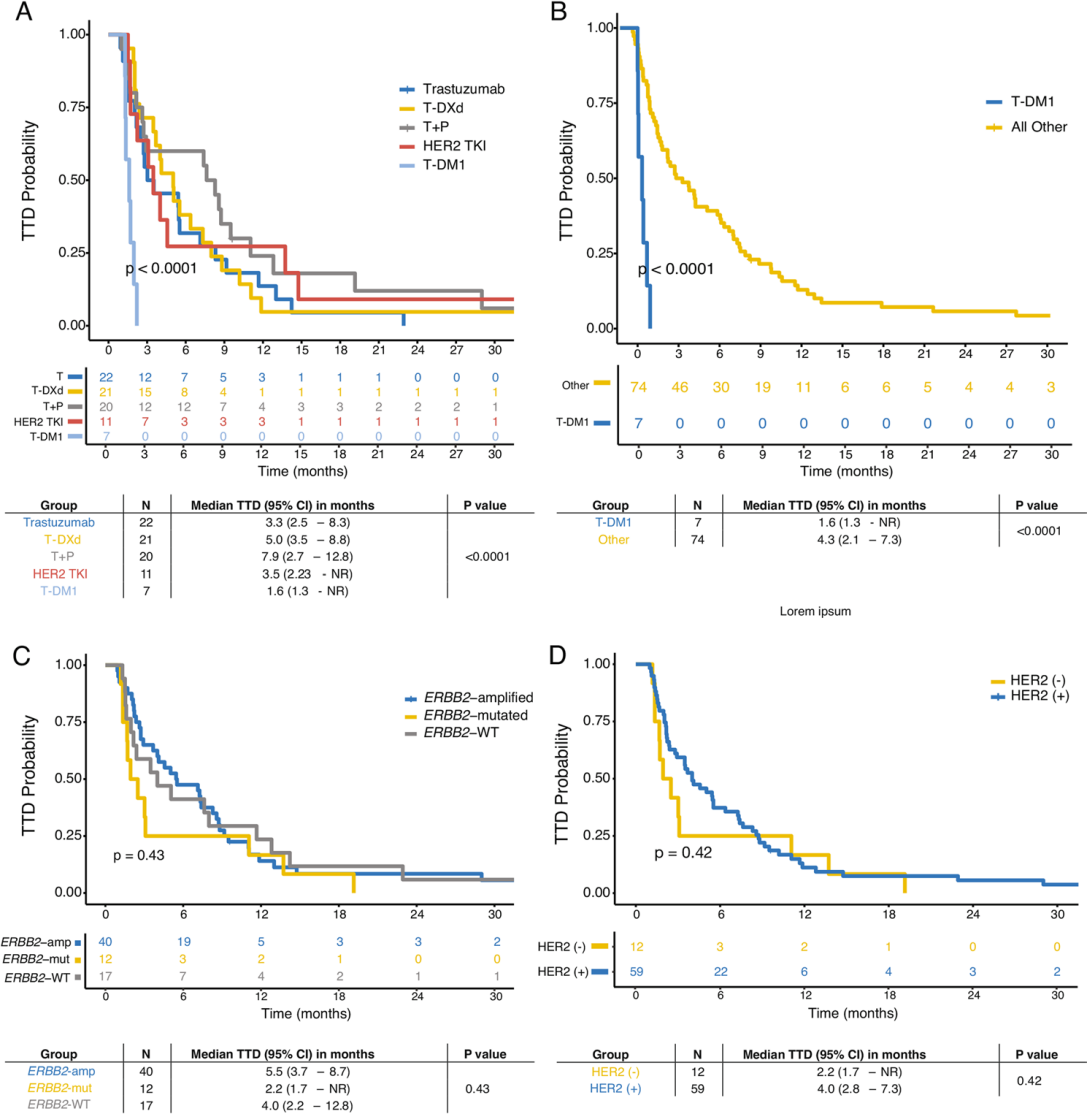
Clinical Activity of HER2-directed Treatments Excluding First-line Therapies. Kaplan-Meier curves showing (**a**) the time to treatment discontinuation (TTD) for each HER2-directed agent administered to patients in the study cohort, excluding first-line systemic treatments, (**b**) the TTDs for patients receiving ado-trastuzumab emtansine (T-DM1) compared to the TTDs for all other HER2-directed treatments in aggregate, (**c**) TTD for 2nd line therapies and beyond stratified by *ERBB2* NGS status, and (**d**) TTD for 2nd line therapies and beyond stratified by HER2 IHC/ISH status. TTDs corresponding to administration of investigational products (IP) were excluded given low overall numbers (*N* < 5 administrations)

Among patients for which time to treatment discontinuation (TTD) data were available (*n* = 81), the median TTD was longest for T + P at 7.9 months (95% CI: 2.7–12.8), followed by T-DXd (5.0 months)(Fig. 3A). TTD for T-DM1 was significantly shorter than for all other HER2-directed agents at 1.6 months (*p* < 0.0001; Fig. 3B). Among genetically-defined cohorts, TTD was numerically longer for *ERBB2*-amplified and *ERBB2*-wild-type tumors (5.5 and 4.0 months, respectively) compared to *ERBB2*-mutated tumors (2.2 months), though this was not statistically significant (Fig. 3C). TTD was also numerically but non-significantly longer for tumors that had high levels of protein expression of HER2 as assessed by IHC (IHC 3 + or IHC 2+/ISH+) compared to HER2-intermediate, low, or non-expressing tumors (IHC 2+/ISH-, 1+, or 0+) (4.4 vs. 2.1 months, respectively) (Fig. 3D).

The growth modulation index (GMI) compares the TTD of current therapy to the previous line of therapy, with values greater than or equal to 1.33 considered to be a significant improvement on the antecedent therapy [14]. Among the 79 GMI evaluable patients, T + P achieved the highest rate of a positive GMI (53% of cases) (Table 2). Trastuzumab combined with chemotherapy, T-DXd, HER2 TKIs, and HER2 investigational agents achieved this at 24–36% rates. By contrast, no patient on T-DM1 reached this threshold. The majority of patients who achieved a GMI ≥ 1.33 stayed on their respective HER2 regimen for at least 6 months (83%; 19/23), including all 9 patients who were treated with T + P. 39% (9/23) of this group remained on their HER2 regimen for over 12 months (Supplementary Table 4).

Twenty-six patients received two or more lines of HER2-directed therapy. In this serially treated cohort, 24% (6/25) had a GMI ≥ 1.33 to their second HER2 regimen, compared to their first. In addition, 16% (4/25; 1 biliary tract cancer [BTC], 1 CRC, and 2 GEC patients) had a TTD of at least 6 months on their second HER2 regimen (Table 3). Three of these patients received T-DXd after initial monoclonal antibody-based treatment (T + P or T alone). For example, one patient (Study ID 04) with BTC achieved an 11-month tumor reduction to T + P before progression and then experienced disease stability on T-DXd for over 2 years (Supplementary Fig. 2A). Another patient (Study ID 20) with CRC initially received T + P, showing only a brief response, but subsequently achieved prolonged disease control on T-DXd for nearly one year (Supplementary Fig. 2B). By contrast, among six patients who received HER2-directed therapy post-T-DXd, only one achieved disease stability beyond 6 months, via an investigational HER2 agent (Supplementary Table 5).

**Table 3:** Patients deriving clinical benefit from serial HER2-directed therapy

Drug (Line of Treatment)*	Prior Treatment	TTD (months)	Best Response	GMI vs. Prior Treatment
Study ID O.04 – BTC				
HER2 TKI (2nd)	Chemotherapy^1^	1.7	PD	0.55
T + P (3rd)	HER2 TKI	11.0	RR	6.46
T-DXd (4th)	T + P	37.2	RR	3.37
Study ID O.20 - CRC				
T + P (5th)	Chemotherapy^2^	2.9	PD	0.26
T-DXd (6th)	T + P	11.9	RR	4.06
Study ID G.54 - Esophageal				
Trastuzumab (1st)	None	21.0	RR	N/A
T + P (4th)	Pembrolizumab	7.4	RR	2.85
HER2 TKI (5th)	T + P	14.8	RR	2.00
Study ID G.58 - Gastric				
Trastuzumab (1st)	None	7.4	RR	N/A
T-DXd (2nd)	Trastuzumab	10.2	SD	1.37

*RR* radiographic reduction, *SD* disease stability, *PD* progressive disease, *N/A* not applicable, *BTC* biliary tract cancer, *CRC* colorectal cancer, *TKI* tyrosine kinase inhibitor, *T + P* trastuzumab plus pertuzumab, *T-DXd* trastuzumab deruxtecan, *TTD* time to treatment discontinuation, *GMI* growth modulation index, *G* Gastric/GEJ/Esophageal, *O* Other histologies

*Intervening non-HER2-directed lines of therapy are not shown

^1^Gemcitabine + nab-paclitaxel

^2^Irinotecan + bevacizumab

An exploratory evaluation of co-mutations was performed for 57 patients with available next-generation sequencing data. *TP53* was the most frequently co-mutated gene, present in 83% of patients. *APC* mutations were observed in 21% of cases and were restricted to CRC and small bowel adenocarcinomas as expected (Supplementary Fig. 3). Other recurrent alterations were noted in cell cycle regulating genes such as *CDKN2A* (19%), *CCNE1* (16%), and *CDKN2B* (7%). *KRAS* genotype was assessed in 60 patients, with 15% (9/60) harboring *KRAS* mutations and 85% being wild-type. All 4 patients with TTD ≥ 24 months to a HER2 agent (1 pancreatic cancer, 2 CRC, and 1 BTC patient) had *KRAS* wild-type disease (Supplementary Table 4), although two patients (1 GEJ and 1 CRC) with *KRAS* mutations nevertheless achieved prolonged disease benefit to a HER2 TKI (TTD: 14.7 months) and T + P (TTD: 12.8 months), respectively. Otherwise, there was no clear correlation between co-mutations and clinical benefit.

## Discussion

The multitude of available HER2-directed agents, both on- and off-label, that have activity in GI cancers suggests that numerous possible treatment pathways can be deployed for this patient population. We describe here a single-institution’s experience that illustrates key principles to guide real-world clinical strategy. Most importantly, we identify a cohort of patients who experienced multi-year benefit from serial lines of HER2 drugs, indicating that early and frequent integration of various HER2 assets into treatment can lead to prolonged clinical benefit.

Second, despite not having an FDA label for gastrointestinal cancers, we find that the chemotherapy-free combination of trastuzumab plus pertuzumab showed the highest real-world time to treatment discontinuation (median of 7.9 months) among the regimens utilized. Even two *KRAS*-mutated colorectal cancer patients derived significant benefit from this regimen (TTD of 9.5 months and 12.8 months, respectively; Supplementary Table 4), despite *KRAS*-mutated colorectal cancer showing greatly reduced benefit to T + P relative to *KRAS-*wild type tumors in a phase 2 study [15]. Additional investigation is needed to better define the role of dual-epitope strategies, whether as a combination of two separate monoclonal antibodies or via a bispecific antibody such as zanidatamab, which was recently approved by the FDA for HER2 + biliary tract cancers [16].

Third, our study highlights the critical nature of multimodal HER2 testing in GI malignancies. Although nearly all (25/26; 96%) of NGS-amplified cases were positive by traditional IHC/ISH criteria, only 66% of HER2-positive cases by IHC showed *ERBB2* amplification. These data align with a prior study reporting a high negative agreement (98.4%) but limited positive agreement (56.2%) between *ERBB2* amplification and IHC/ISH testing specifically in gastroesophageal cancers [12]. Importantly, the observed IHC-positive but NGS-wild-type discordance was consistent across both major NGS platforms used in our cohort (Supplementary Table 3), suggesting that this discordance reflects tumor biology – potentially focal or heterogeneous *ERBB2* amplification that falls below targeted NGS detection thresholds – rather than an assay-specific limitation. All advanced gastrointestinal cancers should therefore undergo HER2 IHC/ISH testing in addition to next-generation sequencing to identify anti-HER2 therapeutic candidates. IHC data may also have prognostic implications; patients in our cohort with higher HER2 expression (3 + by IHC or 2 + by IHC and ISH+) had numerically longer albeit non-statistically significant TTD relative to those with lower HER2 expression, in-line with previously published clinical trial data [17, 18]. Our findings also have practical implications for clinical settings in which NGS is not routinely available or reimbursed. Because IHC/ISH-based testing identified a substantial proportion of anti-HER2 candidates that were missed by NGS in our cohort, and because 93% of NGS-amplified cases in our cohort were also IHC 3+, our findings suggest HER2 IHC with reflex ISH represents a reasonable standalone biomarker strategy for identifying GI cancer patients who may benefit from anti-HER2 therapy where access to NGS is limited. NGS provides complementary information, particularly in the detection of activating *ERBB2* point mutations, but is not strictly required to identify the majority of HER2-positive candidates for treatment.

The limitations of this study include its retrospective design, heterogenous patient population, and the single-institution nature, which limits the ability to draw definitive conclusions. An additional limitation includes the absence of formal inter- and intra-observer concordance data, which are not captured as part of routine clinical diagnostic reporting, and the modest per-platform sample sizes in the NGS concordance analysis. We also note that HER2 scoring criteria for non-gastroesophageal GI tumors were not uniformly standardized during much of the study period, which may contribute to interpretive variability across tumor types. Despite these constraints, these findings support the early and continuous integration of HER2-directed therapies into treatment paradigms, guided by multimodal diagnostics and tailored to individual molecular profiles. Future prospective trials are warranted to clarify optimal sequencing, define a potential greater role for dual HER2 antibody or bispecifics, and further refine biomarker-driven approaches in GI oncology.

## Supplementary Information

Supplementary Material 1.

## Data Availability

The data supporting the findings of this study are available within the article and its supplementary files. The R scripts used to generate the plots in this study are available upon request.
