# Supplementary material for: Clinical benefit of various HER2-directed regimens in gastrointestinal malignancies: a retrospective cohort study
Source: BMC Cancer. 2026 Jun 10;26:983. doi: 10.1186/s12885-026-16309-3 (PMC13474698; doi:10.1186/s12885-026-16309-3)
Supplement: Supplementary file 1 [file 12885_2026_16309_MOESM1_ESM.pdf]

I. SUPPLEMENTARY TABLES

Supplementary Table 1. Full list of treatments received as 1st, 2nd, and 3rd+ lines of therapy

Line of Therapy	Treatment Received (Frequency of Use)
**1**	FOLFOX+trastuzumab (26), capecitabine+carboplatin+trastuzumab (8) CAPOX+trastuzumab (5), FOLFOX+trastuzumab+pembrolizumab (5) gemcitabine+capecitabine (5), XELOX+bevacizumab (4), carboplatin+paclitaxel+trastuzumab (4), gemcitabine+cisplatin (4), FOLFIRI (3), FOLFOX (3), capecitabine+carboplatin+bevacizumab (3), CAPOX+trastuzumab+pembrolizumab (2), FLOT+trastuzumab (2), FOLFIRINOX (2), FOLFOX+bevacizumab (2), FOLFOXIRI (2), XELOX (2), capecitabine (2), carboplatin+docetaxel+trastuzumab (2), gemcitabine+nab-paclitaxel (2), FOLFIRI+bevacizumab (1), FOLFIRI+panitumumab (1), FOLFIRI+trastuzumab (1), FOLFOX+docetaxel (1), capecitabine+carboplatin (1), capecitabine+cisplatin (1), capecitabine+lapatinib (1), capecitabine+trastuzumab (1), carboplatin+5FU+trastuzumab (1), carboplatin+nabpaclitaxel+trastuzumab (1), carboplatin+paclitaxel (1), gemcitabine+cisplatin+nab-paclitaxel (1), gemcitabine+cisplatin+trastuzumab (1), irinotecan (1), nab-paclitaxel+oxaliplatin+trastuzumab (1)
**2**	FOLFOX+trastuzumab (8), paclitaxel+ramucirumab (6), paclitaxel+trastuzumab (6), pembrolizumab (6), trastuzumab deruxtecan (6), FOLFIRI+trastuzumab (5), capecitabine+trastuzumab (3), FOLFIRI (2), ado-trastuzumab emtansine (2), capecitabine (2), gemcitabine+cisplatin (2), gemcitabine+nabpaclitaxel (2), irinotecan+panitumumab (2), paclitaxel+ramucirumab+trastuzumab (2), trastuzumab+pertuzumab (2), trifluridine+tiparicil (2), 5FU (1), Investigational product (1), FOLFIRI+bevacizumab (1), FOLFOX (1), FOLFOX+nivolumab (1), FOLFOX+trastuzumab+pembrolizumab (1), XELIRI+bevacizumab (1), XELIRI+cetuximab (1), capecitabine+bevacizumab (1), capecitabine+cisplatin+docetaxel+ramucirumab (1), carboplatin+5FU+trastuzumab (1), carboplatin+paclitaxel (1), CD8 TIL therapy (1), gemcitabine (1), gemcitabine+capecitabine (1), irinotecan (1), irinotecan+bevacizumab (1), irinotecan+cetuximab (1), nab-paclitaxel+capecitabine+trastuzumab (1), neratinib (1), oxaliplatin+bevacizumab (1), pLADD immunotherapy (1), ramucirumab (1), tucatinib+trastuzumab (1)
**3**	Trastuzumab+pertuzumab (11), irinotecan (4), pembrolizumab (3), trastuzumab deruxtecan (3), 5FU (2), FOLFIRI+trastuzumab (2), FOLFOX (2), gemcitabine+nab-paclitaxel (2), irinotecan+trastuzumab (2), paclitaxel+ramucirumab (2), trastuzumab (2), FOLFIRI+cetuximab (1), FOLFOX+bevacizumab (1), FOLFOX+trastuzumab (1), ado-trastuzumab emtansine (1), ado-trastuzumab emtansine+pembrolizumab (1), capecitabine+bevacizumab (1), capecitabine+trastuzumab (1), carboplatin+nab-paclitaxel+trastuzumab (1), carboplatin+paclitaxel (1), carboplatin+paclitaxel+trastuzumab (1), investigational product (1), docetaxel (1), docetaxel+ramucirumab (1), gemcitabine+cisplatin+durvalumab (1), irinotecan+cetuximab (1), neratinib (1), nivolumab (1), paclitaxel (1), paclitaxel+ramucirumab+trastuzumab (1), paclitaxel+trastuzumab (1), pembrolizumab+trastuzumab (1), trastuzumab+gemcitabine+cisplatin (1), trastuzumab+lapatinib (1), trifluridine-tiparicil (1)
**4**	Trastuzumab deruxtecan (5), gemcitabine+nab-paclitaxel (3), trastuzumab+pertuzumab (3), FOLFIRI (2), FOLFIRI+trastuzumab (2), 5FU+lapatinib (1), FOLFOX (1), ado-trastuzumab emtansine (1), capecitabine+panitumumab (1), irinotecan+bevacizumab (1), nivolumab+regorafenib (1), paclitaxel (1), paclitaxel+ramucirumab (1), panitumumab (1), pembrolizumab (1), pembrolizumab+trastuzumab (1), pembrolizumab+trastuzumab+pertuzumab (1), trastuzumab (1), trastuzumab+5'FU (1), trastuzumab+nivolumab (1), trifluridine+tiparicil (1)
**5**	Trastuzumab deruxtecan (4), trastuzumab+pertuzumab (3), FOLFIRI (2), Investigational product (1), FOLFIRI+nivolumab (1), FOLFIRI+trastuzumab (1), XELOX+panitumumab (1), ado-trastuzumab emtansine (1), capecitabine+trastuzumab (1), lapatinib+capecitabine (1), tucatinib+pembrolizumab (1)
**6**	Trastuzumab deruxtecan (3), FOLFIRI+trastuzumab+nivolumab (1), bevacizumab+capecitabine+IT trastuzumab+tucatinib (1), capecitabine+XRT (1), capecitabine+pembrolizumab+tucatinib (1), gemcitabine+nab-paclitaxel (1), neratinib (1), trastuzumab+tucatinib (1)
**7**	XELOX+trastuzumab (1), ado-trastuzumab emtansine (1), elimusertib+pembrolizumab (1), irinotecan+5FU (1), liposomal irinotecan+5FU (1), trastuzumab+tucatinib (1), tucatinib (1)
**8**	FOLFOX (1), capecitabine+bevacizumab (1), ramucirumab (1)
**9**	Investigational product (1)

Supplementary Table 2. HER2 testing results in cases without HER2 IHC testing

Study ID	Histology	*ERBB2* alteration	*ERBB2* ISH Status
g.15	GEJ adenocarcinoma	Wild type	Positive
g.56	Gastric adenocarcinoma	Wild type	Positive
o.4	Biliary tract cancer	Amplification	
o.9	Pancreas adenocarcinoma	Amplification	
o.17	Biliary tract cancer	Amplification	
o.21	Biliary tract cancer	Amplification	
o.25	Biliary tract cancer	Amplification	Positive
o.37	Colorectal adenocarcinoma	Amplification	
o.38	Biliary tract cancer	Amplification	Positive

GEJ = gastroesophageal junction adenocarcinoma.

Supplementary Table 3. Concordance between HER2+ status[a] by IHC/ISH and *ERBB2* alterations by NGS platform

NGS Platform	N	*ERBB2* amplified	*ERBB2* mutated	*ERBB2* WT
Foundation CDx	23	13 (57%)	0 (0%)	10 (43%)
STAMP	13	9 (69%)	0 (0%)	4 (31%)
Other[b]	5	5 (100%)	0 (0%)	0 (0%)
**All platforms**	**41**	**27 (66%)**	**0 (0%)**	**14 (34%)**

[a] HER2+ defined as IHC 3+, or IHC 2+ with positive ISH, consistent with ToGA/CAP scoring criteria. Foundation CDx versus STAMP amplification detection rate: Fisher exact test p = 0.50.

[b] "Other" includes commercially available targeted sequencing panels each used for a single patient (Biocept, Guardant, Labcorp, Northshore Health, Oncotype MAP, STRATA).

Abbreviations: IHC: immunohistochemistry. ISH: *in situ* hybridization. NGS: next-generation sequencing. Foundation CDx = Foundation Medicine FoundationOne CDx. STAMP = Stanford Solid Tumor Actionable Mutation Panel.

Supplementary Table 4. Tumor and treatment features associated with GMI ≥ 1.33 to HER2-directed therapies (N=23)

Study ID	Agent	Tx Line	Histology	HER2 IHC	HER2 ISH	HER2 NGS	KRAS Status	TTD (months)	Best Response	GMI
O.1	Trastuzumab	4	Small Bowel	0	(-)	Mutated	Mutated	3.0	NA	1.39
O.13	HER2 TKI	6	Pancreas	0		Mutated	WT	3.1	PD	1.80
O.41	T-DXd	4	Small Bowel	2+	(+)	WT	WT	3.5	SD	4.82
G.56	T-DXd	2	Gastric		(+)	WT	Mutated	5.1	SD	1.81
O.15	IP	2	CRC	3+		Amplified	WT	7.3	SD	2.80
G.54	T+P	4	Esophagus	3+		Amplified	Mutated	7.4	RR	2.85
O.19	T+P	2	CRC	2+	(+)	WT	WT	7.6	SD	1.69
O.21	T+P	3	BTC			Amplified	WT	8.3	SD	4.34
G.29	Trastuzumab	3	Esophagus	3+				8.3	SD	5.50
O.39	T+P	4	BTC	3+		Amplified	Mutated	8.7	SD	1.90
O.14	Trastuzumab	2	CRC	3+		Amplified	WT	9.2	RR	1.37
O.40	T+P	3	CRC	3+	(+)	Amplified	WT	9.5	RR	2.78
G.58	T-DXd	2	Gastric	2+	(+)			10.2	SD	1.37
O.4	T+P	3	BTC			Amplified	WT	11.0	RR	6.46
O.26	Trastuzumab	2	BTC	2+	(+)	WT	WT	11.7	RR	1.46
O.20	T-DXd	6	CRC	3+		Mut + Amp	WT	11.9	RR	4.06
O.32	T+P	3	CRC	3+		WT	Mutated	12.8	SD	10.83
O.33	HER2 TKI	3	BTC	2+	(-)	Mutated	WT	13.7	RR	5.50
G.15	Trastuzumab	2	GEJ		(+)	WT	WT	14.2	RR	13.12
G.54	HER2 TKI	5	Esophagus	3+		Amplified	Mutated	14.8	RR	2.00
G.45	Trastuzumab	2	Gastric	2+	(+)	WT	WT	22.9	SD	4.68
O.6	T+P	5	CRC	3+		Amplified	WT	29.0	RR	12.44
O.4	T-DXd	4	BTC			Amplified	WT	37.2	RR	3.37
O.23	T+P	3	Pancreas	3+		Amplified	WT	41.0	SD	19.79
O.22	HER2 TKI	2	CRC	3+	(+)	WT	WT	65.6	SD	16.23

Table is arranged in order of ascending time to treatment discontinuation (TTD). Blank cells represent missing/unknown data.

Abbreviations: GMI: growth modulation index. RR: radiographic reduction. IHC: immunohistochemistry. ISH: in situ hybridization. NGS: nextgeneration sequencing. WT: wild type. Mut + Amp: Co-amplified and mutated. GEJ: gastroesophageal junction. BTC: biliary tract cancer. CRC: colorectal adenocarcinoma. TKI: tyrosine kinase inhibitor. T-DXd: trastuzumab deruxtecan. T+P: trastuzumab plus pertuzumab. IP: investigational product.

Supplementary Table 5. Outcomes of Anti-HER2 Agents after Progression on T-DXd (N=6)

Agent	Number of Uses	TTD ≥ 6 months (%)
HER2 TKIs	4	0 (0/4)
T-DM1	3	0 (0/4)
Trastuzumab	1	0 (0/1)
T+P	1	0 (0/1)
IP	1	100 (1/1)

T-DXd: trastuzumab deruxtecan. TKI: tyrosine kinase inhibitor. T-DM1: ado-trastuzumab emtansine. T+P: trastuzumab plus pertuzumab. IP: investigational product. TTD: time to treatment discontinuation.

II. SUPPLEMENTARY FIGURES:

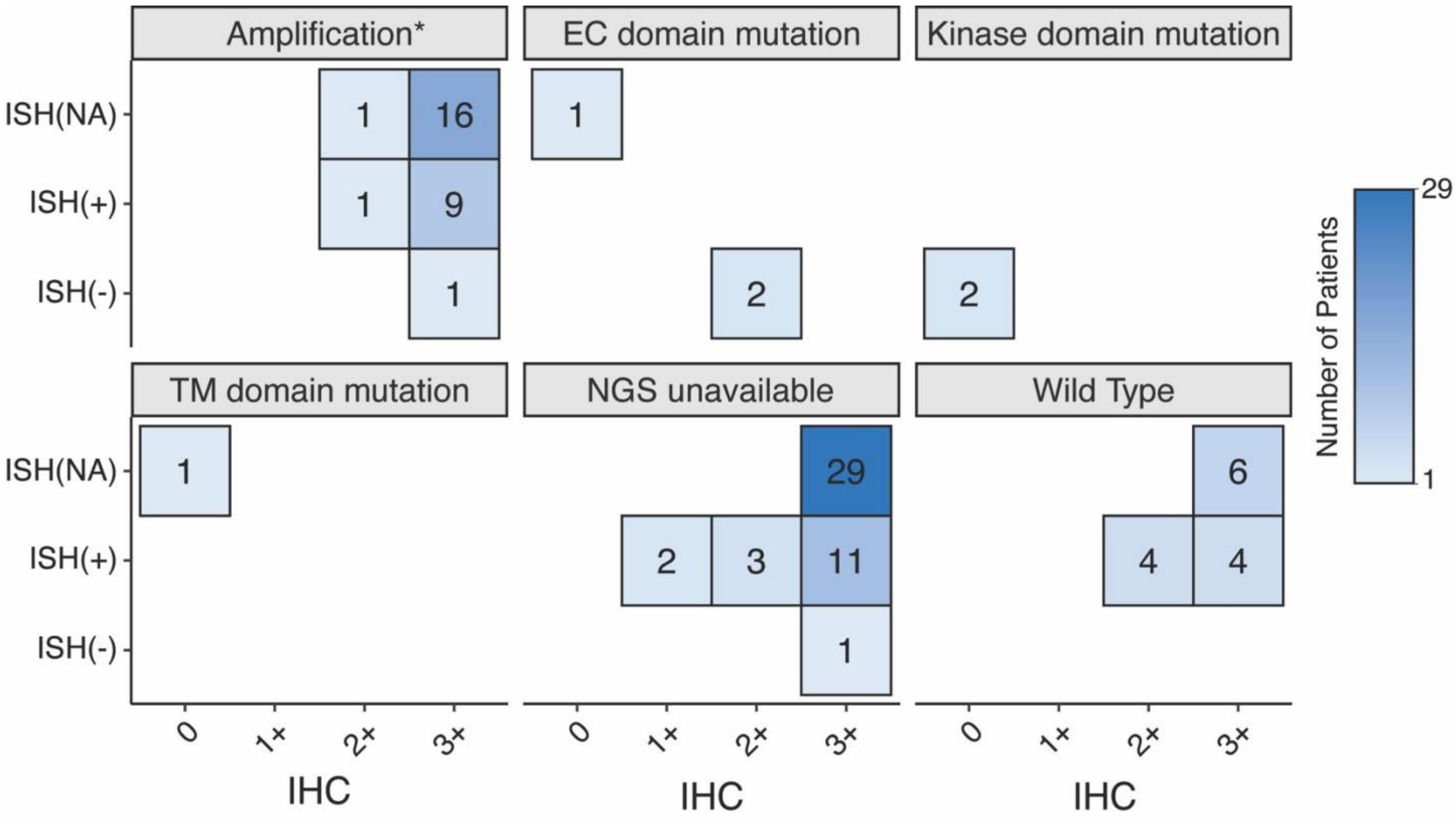

Supplementary Figure 1. Intersection of HER2 testing results within the study cohort. Each gray-colored panel indicates the *ERBB2* alteration determined by NGS testing (e.g., amplification, wild type). Within each labeled panel, blue-shaded boxes represent the number of cases with a given *ERBB2* genotype that also exhibit a specific HER2 IHC or ISH result. Darker blue boxes represent a higher number of cases. EC: extracellular. TM: transmembrane. *Two patients with tumors harboring both an amplification and an ERBB2 S310F variant were included in this category (both were IHC 3+).

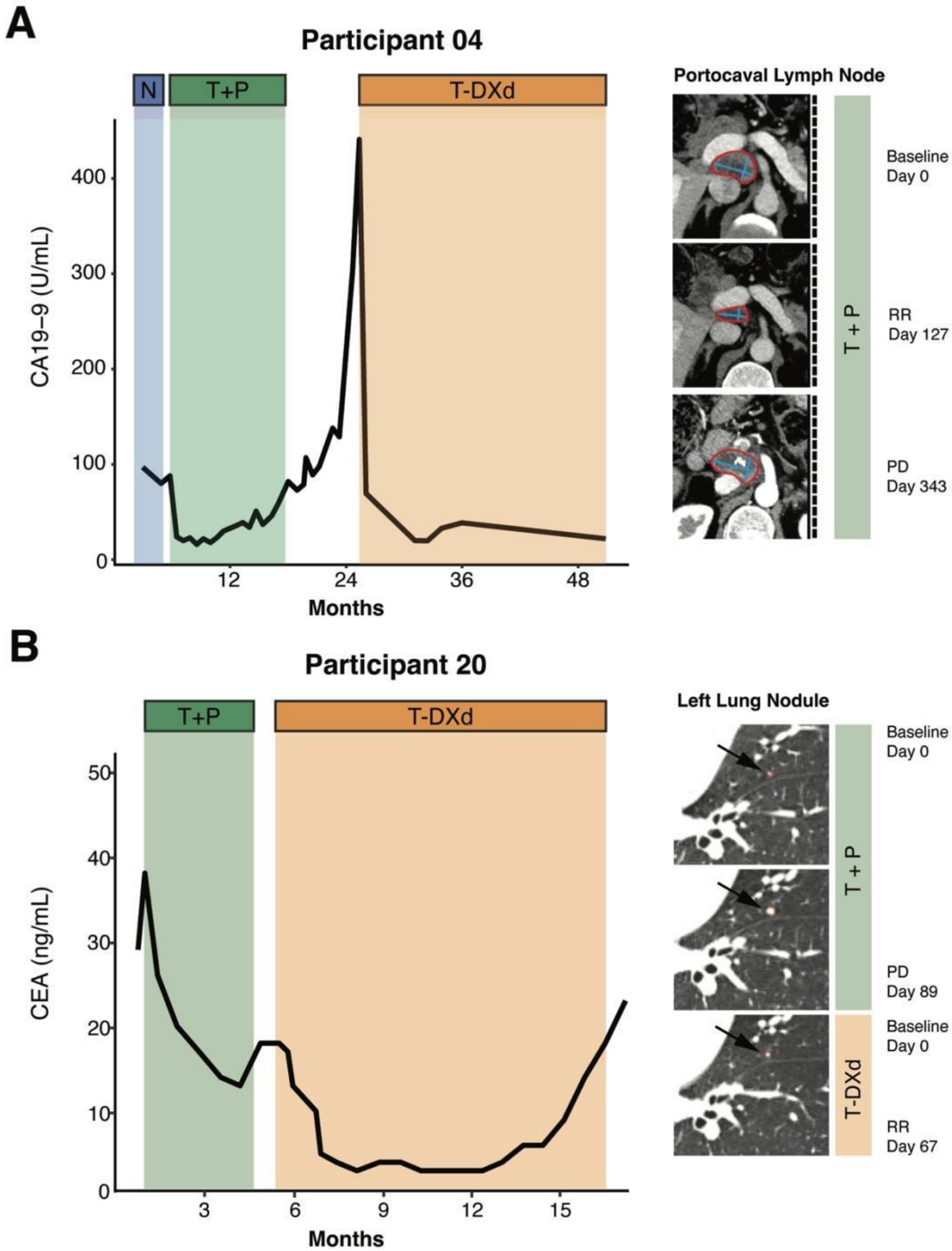

Supplementary Figure 2. Clinical Benefit of Serial HER2-Directed Agents. (A) Plot of Participant 04's CA19-9 levels over the course of receiving HER2-directed therapies, juxtaposed with crosssectional computed tomographic (CT) images showing changes in a portocaval lymph node in response to treatment. (B) Plot of Participant 20's CEA levels over the course of receiving HER2-directed therapies along with cross-sectional CT images of their lung nodule and its response to HER2-directed therapy. N = neratinib. T + P = trastuzumab + pertuzumab. T-DXd = trastuzumab deruxtecan. RR: radiographic reduction. PD: progressive disease.

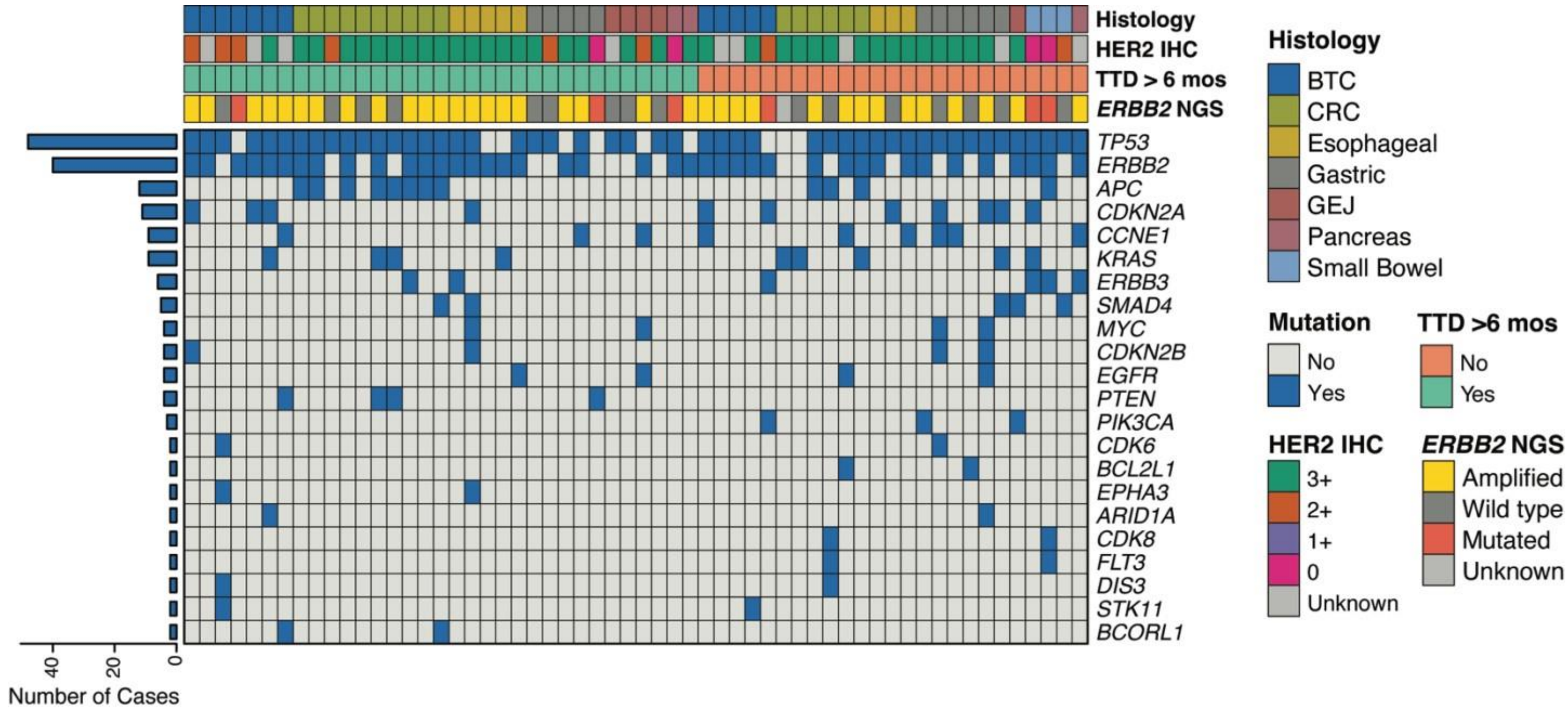

Supplementary Figure 3. Molecular co-alterations in patients with NGS data. The top panels above the co-mutation plot show the distribution of tumor histologies, HER2 IHC scores, and *ERBB2* alterations by NGS, along with an indicator of whether each patient achieved a six-month time to treatment discontinuation (TTD) to any HER2-targeted treatment regimen. Each column represents 1 patient. The bar graphs and percentages on left represent the numbers of patients whose tumors harbored a mutation in the corresponding row's gene. IHC: immunohistochemistry. NGS: nextgeneration sequencing. Mos: months.
